# A global view on carbapenem-resistant *Acinetobacter baumannii*

**DOI:** 10.1128/mbio.02260-23

**Published:** 2023-10-26

**Authors:** Carina Müller, Sandra Reuter, Julia Wille, Kyriaki Xanthopoulou, Danuta Stefanik, Hajo Grundmann, Paul G. Higgins, Harald Seifert

**Affiliations:** 1 Institute for Medical Microbiology, Immunology and Hygiene, Faculty of Medicine and University Hospital Cologne, University of Cologne, Goldenfelsstr, Cologne, Germany; 2 German Center for Infection Research (DZIF), Partner Site Bonn-Cologne, Cologne, Germany; 3 Institute for Infection Prevention and Hospital Epidemiology, Medical Centre–University of Freiburg, Freiburg, Germany; Institut Pasteur, Paris, France

**Keywords:** *Acinetobacter baumannii*, carbapenem resistance, molecular epidemiology, genome analysis, carbapenemase, international clone

## Abstract

To give an update on the molecular epidemiology and global distribution of carbapenemase encoding genes, we subjected 313 carbapenem-resistant *Acinetobacter baumannii* isolated from 114 study centers in 47 countries in five world regions, Africa, Asia, Europe, Latin America, and North America, to whole genome sequencing. Numbers of isolates investigated were proportional to the population size of the contributing countries. Molecular epidemiology was investigated using seven-loci and core genome multilocus sequence typing, whole-genome single nucleotide polymorphism phylogenies, and the intrinsic *bla*_OXA-51-like_ variant. Carbapenemase encoding genes were identified by multiplex PCR and ResFinder. Among the total of 313 isolates, 289 (92.3%) were assigned to *A. baumannii* international clones (IC) IC1–IC8. IC2 predominated with 196 isolates (62.6%) and was spread worldwide, followed by IC5 with 44 isolates (14.1%) mainly confined to Latin America. Six isolates (1.9%) originating from Belgium, Egypt, Italy, and Pakistan represent the novel IC9. Acquired OXA-type carbapenemase genes were found in 300 (96%) isolates with *bla*_OXA-23-like_ and *bla*_OXA-40-like_ predominating, which constitutes a significant increase compared to our findings from 2010. Metallo-beta-lactamases were rare with seven isolates (2.2%). The distribution of ICs and carbapenemase determinants can vary widely among different geographical regions.

**IMPORTANCE:**

Carbapenem-resistant *Acinetobacter baumannii* are of increasing public health importance, as they are resistant to last-line antibiotics. International clones with well-characterized resistance genes dominate globally; however, locally, other lineages with different properties may be of importance to consider. This study investigated isolates from a broad geographic origin from 114 hospitals in 47 countries and from five world regions ensuring the greatest possible diversity in an organism known for its propensity for clonal epidemic spread and reflecting the current global epidemiology of carbapenem-resistant *A. baumannii*. In Latin America, a lineage different from other geographic regions circulates, with a different resistance gene profile. This knowledge is important to adjust local infection prevention measures. In a global world with migration and increasing use of antimicrobials, multidrug-resistant bacteria will continue to adapt and challenge our healthcare systems worldwide.

## INTRODUCTION

*Acinetobacter baumannii* is an important nosocomial pathogen causing severe infections such as ventilator-associated pneumonia, bloodstream infection, urinary tract infection, meningitis, and wound infection, particularly in critically ill patients (1, 2). The pathogen has become a healthcare challenge worldwide due to its antimicrobial resistance and propensity for clonal spread (3). Furthermore, resistance to desiccation enables the organism to survive on inanimate surfaces for extended periods of time, which contributes to its transmission within the hospital setting, resulting in outbreaks and endemic persistence (1). Its intrinsic and acquired antimicrobial resistance is a severe threat to the successful treatment of *A. baumannii* infections (4). In particular, the increasing carbapenem resistance that has evolved during the last decades deprives us of these front-line antimicrobial agents to treat *A. baumannii* infections (3).

Carbapenem-resistant *A. baumannii* (CRAB) is considered as priority 1 (“critical”) in the WHO priority pathogens list for research, discovery, and development of new antibiotics (5) and has recently been upgraded to an urgent public health threat by the CDC (https://www.cdc.gov/drugresistance/pdf/threats-report/2019-ar-threats-report-508.pdf). In 2017, CRAB was found causing infection in an estimated 8,500 hospitalized patients in the USA, leading to 700 deaths.

Resistance to carbapenems is mainly mediated through acquired OXA-type carbapenem-hydrolyzing class D β-lactamases [oxacillinases (OXAs)], encoded by *bla*_OXA-23-like_, *bla*_OXA-40-like_, *bla*_OXA-58-like_, *bla*_OXA-143-like_, and *bla*_OXA-235-like_ (6–8). Some variants of the intrinsic OXA-51-like carbapenemase confer carbapenem resistance when overexpressed via IS*AbaI* (9, 10). Less frequently, carbapenem resistance in *A. baumannii* is mediated through class B metallo-beta-lactamases (MBLs) such as IMP, NDM, SIM, and VIM (1, 11) and only rarely by class A KPC and GES beta-lactamases (12, 13).

Since *A. baumannii* outbreaks emerged during the last decades, often in association with antimicrobial resistance, the epidemiological relationship within the *A. baumannii* population was the subject of investigations using different molecular typing methods (14). Previously, three clones of closely related *A. baumannii* isolates were mainly responsible for outbreaks in Europe between the 1980s and early 2000s, and these were termed European clones EUI, II, and III (15, 16). More recently, it became clear that the vast majority of CRAB worldwide could be assigned to just a few widespread *A. baumannii* clonal lineages. Using repPCR, a global collection of more than 400 CRAB isolates clustered into eight clonal lineages initially termed worldwide (WW) clones and later adopted the term international clones (IC) 1–8, with IC1–3 corresponding to EUI–EUIII (14, 17).

Two 7-loci multilocus sequence typing (MLST) schemes for *A. baumannii*, referred to as “Oxford” (Ox) and “Pasteur” (Pas) scheme, proved to be valuable tools to analyze the population structure of *A. baumannii* on a global scale (18, 19). Sequence types (STs) determined by MLST clustered into clonal complexes (CCs) corresponding to the ICs previously determined by repPCR (20). Also, the variant of the intrinsic OXA-51-like carbapenemase has been suggested as a suitable marker for the assignment of *A. baumannii* isolates to the international clonal lineages (21).

Since the advent of relatively cheap whole genome sequencing (WGS), this technique has been adopted to investigate the molecular epidemiology of *A. baumannii* and to investigate outbreaks and was shown to give a higher resolution than MLST (22, 23). Although the use of single nucleotide polymorphism (SNP) typing gives the highest resolution, core genome multilocus sequence typing (cgMLST) provides a more stable nomenclature and results that are easier to share with others (24, 25).

The aim of this investigation was to elucidate the current worldwide molecular epidemiology from a contemporary collection of 313 phenotypically confirmed CRAB isolates and the distribution of carbapenemases using a population-based approach. WGS was applied to extract STs^Ox^ and STs^Pas^, cgMLST, *bla*_OXA-51-like_ variants, carbapenemase encoding genes, and capsule locus K as well as outer core locus (OCL) from a global cohort of CRAB isolates. Intra- and interregional clustering was investigated by resolving phylogenetic relationships for the most important international clones.

## RESULTS

### Species confirmation and antimicrobial susceptibility testing

Among the initial cohort of 326 isolates, 313 isolates from 114 study centers in 47 countries were positive for intrinsic *bla*_OXA-51-like_ and confirmed to be *A. baumannii* and resistant to meropenem and imipenem (Tables S1, S2, and S3). The remaining 13 isolates could either not be confirmed as *A. baumannii* (*n* = 2) or tested susceptible or intermediate to imipenem and/or meropenem with E-test (*n* = 11) and were, therefore, excluded.

### Molecular epidemiology

**Fig 1:**
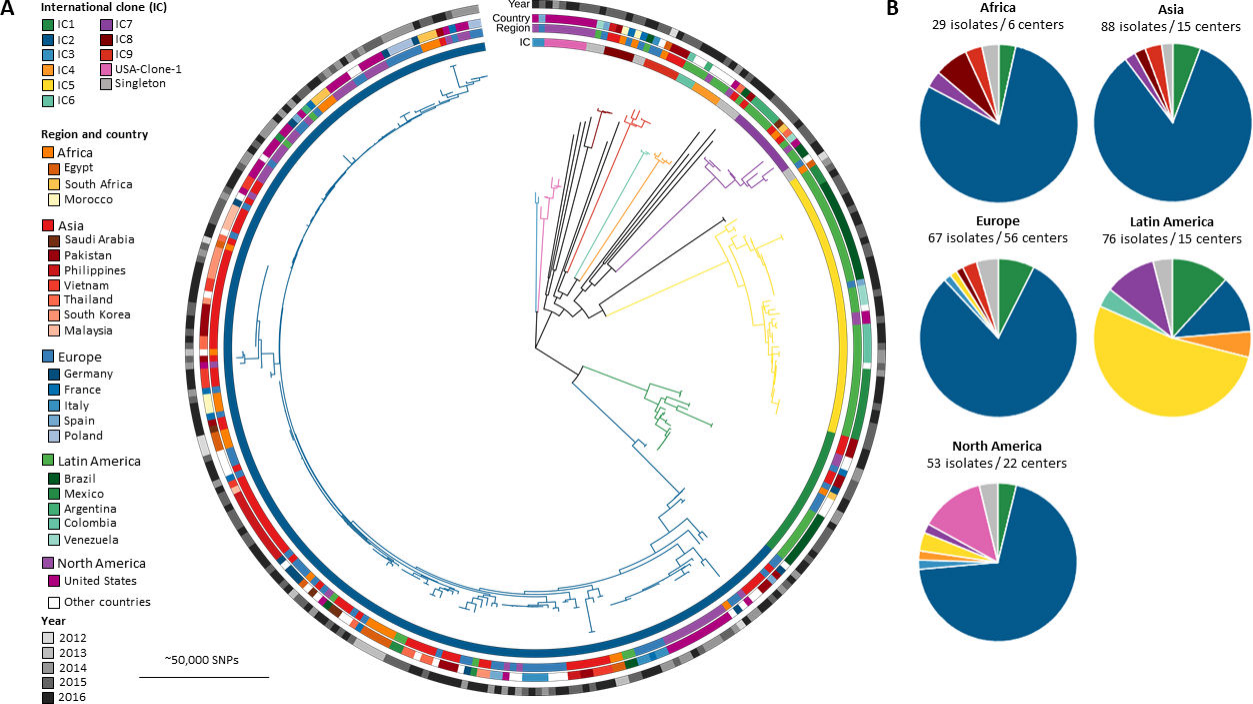
(**A**) Global phylogeny of *A. baumannii* with distribution of isolates and international clones. Midpoint-rooted phylogeny of collected isolates. Global clones are highlighted by colored branches as well as the innermost circular ring. Regions are colored, as are countries with more than five isolates within these regions. Graded gray color scheme for year of sample collection. (**B**) Geographical distribution of ICs of the 313 carbapenem-resistant *A. baumannii* isolates, number of isolates, and contributing centers.

Based on the CCs derived from seven-loci MLST analysis in the “Oxford” and “Pasteur” schemes and the variants of the intrinsic *bla*_OXA-51-like_ carbapenemases, 289 (92.3%) among the total of 313 isolates could be assigned to the established eight ICs, IC1–IC8 (Table 1). We then reconstructed a species-wide phylogeny of all sequenced isolates (Fig. 1A). The geographical distribution of ICs is illustrated in Fig. 1B and presented in Table S1. All metadata of the isolates of the current study are depicted in Table S1.

**TABLE 1:** Geographical distribution of *A. baumannii* international clonal lineages^*a*^

Region (no. of isolates)	IC1	IC2	IC3	IC4	IC5	IC6	IC7	IC8	IC9	No IC
Africa (29)	1 (3.4%)	23 (79.3%)					1 (3.4%)	2 (6.9%)	1 (3.4%)	1 (3.4%)
Asia (88)	5 (5.7%)	74 (84.0%)					2 (2.3%)	2 (2.3%)	3 (3.4%)	2 (2.3%)
Europe (67)	5 (7.5%)	54 (80.6%)	1 (1.5%)		1 (1.5%)			1 (1.5%)	2 (3.0%)	3 (4.5%)
Latin America (76)	9 (11.8%)	8 (10.5%)		4 (5.3%)	41 (54.0%)	3 (4.0%)	8 (10.5%)			3 (4.0%)
North America (53)	2 (3.8%)	37 (69.8%)	1 (1.9%)	1 (1.9%)	2 (3.8%)		1 (1.9%)			9 (17.0%)
Total (313)	22 (7.0%)	196 (62.6%)	2 (0.6%)	5 (1.6%)	44 (14.1%)	3 (1.0%)	12 (3.8%)	5 (1.6%)	6 (1.9%)	18 (5.8%)

^*a*^ IC, international clone; No IC, isolates not assigned to any known IC.

Distinct lineages corresponding to the previously characterized IC1–IC8 are visible, and we identified two additional lineages, which we termed IC9 (red, 1 o’clock) and USA-clone-1 (pink, 12 o’clock; described further down). Clustering by region or country was investigated for the most relevant ICs IC1, IC2, IC5, IC7, and IC9. There was no temporal clustering evident by year of isolation.

**Fig 2:**
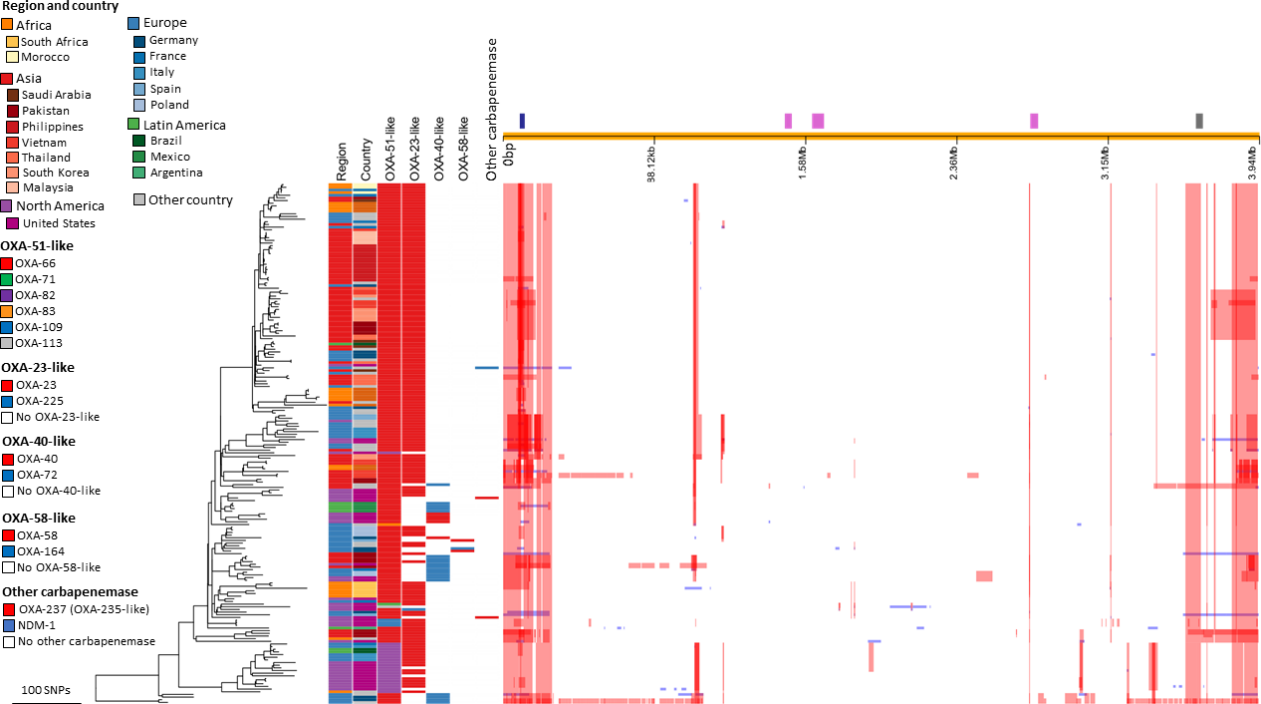
Phylogeny of IC2 with associated carbapenemases and regions of recombination. Colored strips next to the phylogeny show region and country of isolation and presence of various carbapenemases (intrinsic and acquired). Recombination blocks (red) along the chromosome coordinates (top) are highlighted. The capsule K locus (blue) is under recombination, as is the pilus/fimbrial operon (gray), whereas phage loci (pink) are not. Two isolates of ST636 at the base of the tree show larger regions of recombination, corresponding to the fact that this ST shares alleles both with ST1 (three alleles) and ST2 (four alleles).

IC2 constituted the largest and most widely spread clonal lineage with 196 isolates (62.6%) distributed over all participating regions (Fig. 1A and B; Table 1). Most of the IC2 isolates were part of a successful clonal expansion that shows limited diversity. Two isolates with the Pasteur ST636 clustered between IC1 and IC2 suggesting it is a hybrid of ST1 and ST2. This ST is comprised of three ST1 alleles and four ST2 alleles, and analysis together with IC2 isolates showed evidence of large segments of allelic exchange supporting the hybrid genotype (Fig. 2, most basal isolates).

IC5 was the second most frequent IC with 44 isolates (14.1%) and was predominant in Latin America, accounting for 41 of 76 isolates (54%) recovered from this region (Table 1; Fig. S1). IC1 was as widespread over all participating regions as IC2, but with a much lower prevalence, accounting for only 22 isolates (7.0%) (Fig. S2).

Among the 24 remaining isolates that could not be assigned to any established IC, there were six isolates from Egypt (1), Pakistan (3), Belgium (1), and Italy (1) that shared the same sequence type ST85^Pas^ (Table S3). Therefore, we propose calling this internationally spread clone “IC9.”

Among the 18 remaining isolates not assigned to any international clone, a further seven isolates from the USA sharing ST406^Pas^ formed a group of closer related isolates. These isolates originated from four different centers spread among four US states, i.e., Illinois, Indiana, Nebraska, and Wisconsin; therefore, we propose calling this cluster USA-clone-1 (Fig. 1).

When we look at the expanded data set downloaded from Pathogenwatch (https://pathogen.watch/; Table S5), we can see that two countries are contributing a disproportionate amount of genome sequences to the public databases, USA and China (5,026 and 1,017 sequences, respectively). Of note, half of the isolates from China are from one particular study from 2019. Unfortunately, comparatively few sequences (176 sequences) are available from India, and nearly all of them are from a single study. While the database contains old sequences starting from 1930 onwards, 77% of the sequences (6,696 of 8,660) were collected between 2012 and 2022. The dominant international clone is again IC2, making up 65% of the data set (85% among sequences from China), and 15% of sequences cannot be assigned to a described lineage. In about one-fifth of the genome sequences (22%), we did not detect any acquired carbapenemase-encoding genes, indicating that the majority of sequencing is currently directed toward multidrug-resistant isolates.

### Overall distribution of carbapenemase genes

**Fig 3:**
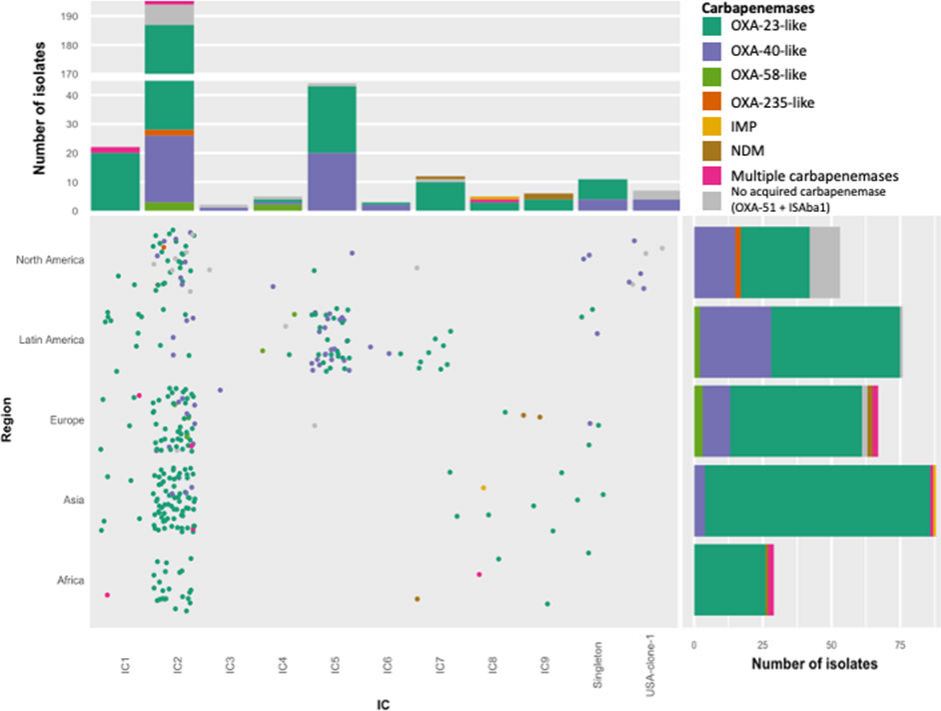
Distribution of carbapenemases across international clones and regions. Top boxplot shows the number of isolates per IC and carbapenemase; note the broken y-axis for non-distortion by overrepresentation of IC2. Jitterplot: visual representation of isolates per IC and region. Tilted boxplot shows the number of isolates per region and carbapenemase. The color scheme is the same across all plots and given in the top right corner.

Investigation with multiplex PCR and ResFinder identified acquired carbapenemase genes in 300 (96%) of the isolates. Fig. 3; Table S4 show the geographical distribution of acquired OXAs and MBLs. The most frequently encountered carbapenemase-encoding genes were *bla*_OXA-23-like_ and *bla*_OXA-40-like_, present in 234 (74.8%) and 56 isolates (17.9%), respectively. Both in Latin and North America, the number of *bla*_OXA-40-like_-positive isolates was considerably higher than in the remaining regions with nearly 32% of all American isolates. This is particularly visible in Latin America, where 46% of all IC5 isolates carried *bla*_OXA-40_. Other carbapenemases such as MBLs were rarely found (*n* = 7, 2.2%). Six isolates (1.9%) harbored *bla*_NDM-1_, four from Europe (Belgium, Italy, and the Netherlands) and two from South Africa, whereas one isolate from the Philippines was positive for *bla*_IMP-26_.

Thirteen isolates (4.2%) that did not possess an acquired carbapenemase gene were positive for IS*Aba1* upstream of *bla*_OXA-51-like_, 10 isolates from the USA (*bla*_OXA-82_, 5; *bla*_OXA-172_, 3; *bla*_OXA-113_, 2), one isolate from Chile (*bla*_OXA-219_), one from the Czech Republic (*bla*_OXA-83_), and one from Spain (*bla*_OXA-65_). Tables S3 and S4 show the specific variants of all carbapenemase genes found. Thirty-two isolates with an acquired carbapenemase-encoding gene were also positive for IS*Aba1* upstream of *bla*_OXA-51-like_. Two novel *bla*_OXA-51-like_ variants were found and submitted to the NCBI Bacterial Antimicrobial Resistance Reference Gene Database, i.e., *bla*_OXA-828_ (MK913672.1) and *bla*_OXA-829_ (MK913673.1).

### Capsule loci

As contributors to virulence and phage susceptibility, we searched for capsule polysaccharide K locus (KL) and outer core OC locus (OCL) types (Table S1). The OCL1 capsule was predominant in IC2 (87% of isolates), but 27 different KL variants were present, with KL2 and KL3 accounting for 20% of the isolates each. In IC1, OCL1 was also predominant (59%), and eight different KL variants were present; interestingly, KL1 was restricted to isolates from Brazil. OCL10 was predominant in IC5 (93%) and IC7 (58%), and OCL6 was predominant in IC9 (83%). The KL variants for IC5 were KL49 and KL9 (23% and 37%, respectively), and in IC7, KL14 and KL81 predominated (41% and 33%, respectively). Overall, we found 37 KL types, with KL2, KL3, and KL9 representing about one third of the types (13%, 13%, and 12%, respectively). In contrast, there were only 10 different O capsule types; however, these were dominated by OCL1 and OCL10 due to their presence in the predominant international clones IC1, IC2, IC5, and IC7.

### Detailed characterization of relevant international clones

We then set to characterize the top five international clones using data from this study as well as additional published genomes for IC5, IC7, and IC9. For a detailed characterization of IC1 and IC2, we mapped the reads to references of the specific lineage and excluded potential recombinogenic regions from the phylogenetic analysis. For IC5 and IC7, we included additional assemblies from Pathogenwatch (Tables S6 and S7) and based the phylogeny on the core genes identified. For IC9, we followed the same approach as for IC1 and IC2 and included additional genome sequences from other studies as identified through Pathogenwatch (Table S8). We then overlaid this with information on the carbapenemase variants, recombination hotspots (for IC1 and IC2), and information on KL and O loci (see above).

Fig. 2 shows the recombination-free phylogeny for the 196 IC2 isolates from this study. IC2 appeared as a quite heterogenous group of isolates with numerous smaller, country- or region-specific clusters within. There were two clades from Asia, one predominantly from the Philippines and Malaysia and the other from Pakistan, South Korea, and Vietnam. There were also smaller clades with isolates predominantly from Europe and the USA. Most isolates (81%) carried *bla*_OXA-23_ in addition to the OXA-51-like *bla*_OXA-66_ variant (87%) (Fig. 2). One particular basal clade found in Brazil, France, Italy, Tunisia, and the USA contains the *bla*_OXA-66_ variant *bla*_OXA-82_ (L167V substitution) which was associated with IS*Aba1*. The recombination hotspots are centered on 250 kb either side of the origin of replication. Regions under recombination are the capsule locus as well as the pilus/fimbrial operon and metabolic pathways. After removing SNPs constituting recombination, the maximum SNP distance including the two ST636 isolates was 331 SNPs difference between isolates.

Similar recombination hotspots corresponding to known phage regions, the capsule locus, as well as an arsenic resistance operon within the multiple-antibiotic resistance region were also found in IC1 (Fig. S2). Within IC1 isolates, there was one particular sublineage with closely related isolates that appears to be expanding in Brazil (Fig. S2).

**Fig 4:**
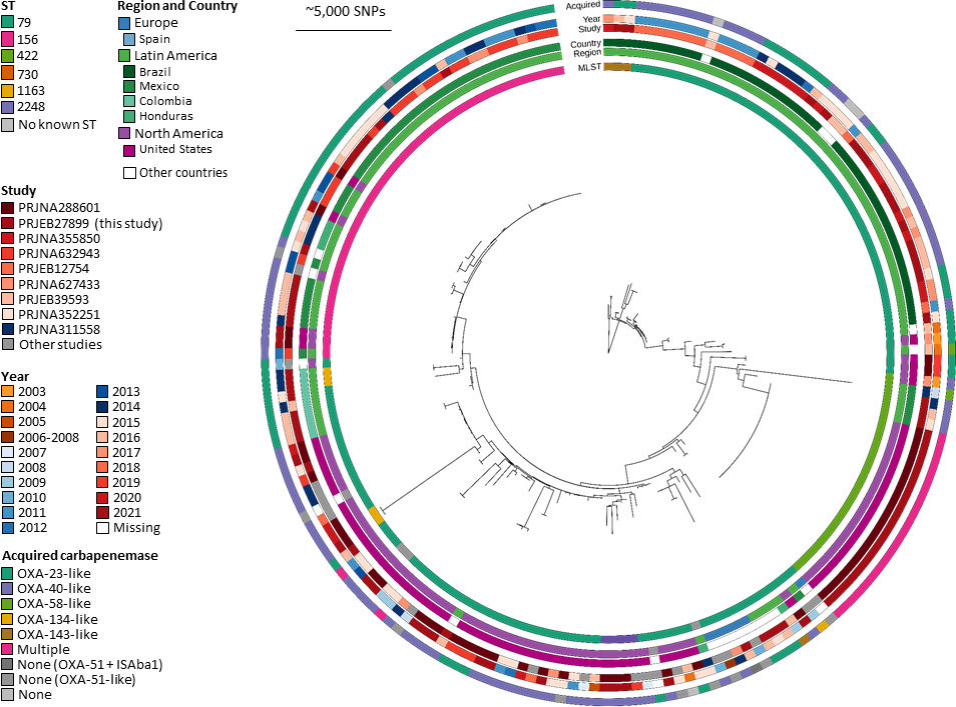
View of IC5. Whole genome maximum likelihood phylogeny of Latin American IC5 based on 44 isolates from this study and 145 genomes from public databases. The innermost circle shows ST, followed by region and country, then study and year to show whether isolates may be part of particular outbreak investigations, and lastly acquired carbapenemases. Several distinct clades with geographic restrictions are visible. A predominance of Latin American isolates is evident, with single exportation events, although a large proportion of isolates are also linked to North America.

For analysis of IC5, we included sequences from 44 isolates from this study and 145 sequences from published genomes. The most basal sequence type is ST730, and ST79 is the main ST within this IC (Fig. 4). From this, ST156, ST422, and ST2248 emerged as coherent clades, ST1163 emerged twice, and there are several unassigned STs. As was already apparent in the worldwide distribution of international clones (Fig. 1; Table 1), IC5 is a prominent Latin American clone; however, based on the expanded data set from published genomes, a considerable number of sequences were from North America. The most basal clade (Fig. 4, 1–3 o’clock) contains isolates from Brazil, with small export events to Bolivia. A second Latin American clade contains isolates from Mexico, Colombia, and Honduras, with reverse export events into the USA and Canada (9–12 o’clock), which is associated with ST156. A loose clade contains isolates from Latin America and Europe, although they are separate and not intermingling (5 o’clock). One clade—presumably an outbreak, as all samples are from one study and the same year and highly clonal—contains isolates exclusively from the USA and can be identified as ST422 (4 o’clock). A second North American clade shows more diversity, with export events into Latin America (6–8 o’clock). The dominant acquired carbapenemases are either OXA-23-like or OXA-40-like, with a tendency for Latin American isolates toward *bla*_OXA-23_, with particular focus of the *bla*_OXA-23_ variant *bla*_OXA-239_ in Mexico, and North American isolates toward *bla*_OXA-40_ (Table S6). Latin American isolates with OXA-40-like genes are also different variants, particularly *bla*_OXA-72_ and *bla*_OXA-253_. The exceptions in these are the export (import) events that are presumably travel associated. The particular US outbreak clade ST422 contains multiple carbapenemases, i.e., a combination of *bla*_OXA-58_, *bla*_OXA-72_, and *bla*_NDM-1_. Comparison of a subset of isolates between WGS and cgMLST showed coherent clustering (Fig. S1).

We also analyzed 139 IC7 sequences (12 sequences from this study and 127 sequences from published genomes; Fig. S3). The most basal ST is ST113, and the main ST is ST25. One particular outbreak (9 o’clock) is characterized by a change of ST25 to ST619. There appears to be several geographically restricted clades. One clade predominantly contains isolates from the USA and Libya (3–6 o’clock), whereas another clade contains mostly Asian isolates from India, China, and Thailand (9–12 o’clock). Latin American isolates are more loosely clustered (between 7 and 8 o’clock). All except two isolates carry *bla*_OXA-64_ as the intrinsic *bla*_OXA-51_ variant, while the predominant acquired carbapenemase is *bla*_OXA-23_. A particular clade within ST113 carries the *bla*_OXA-40-like_ variant *bla*_OXA-253_, whereas other acquired OXA and NDM carbapenemases are not restricted to particular clades.

**Fig 5:**
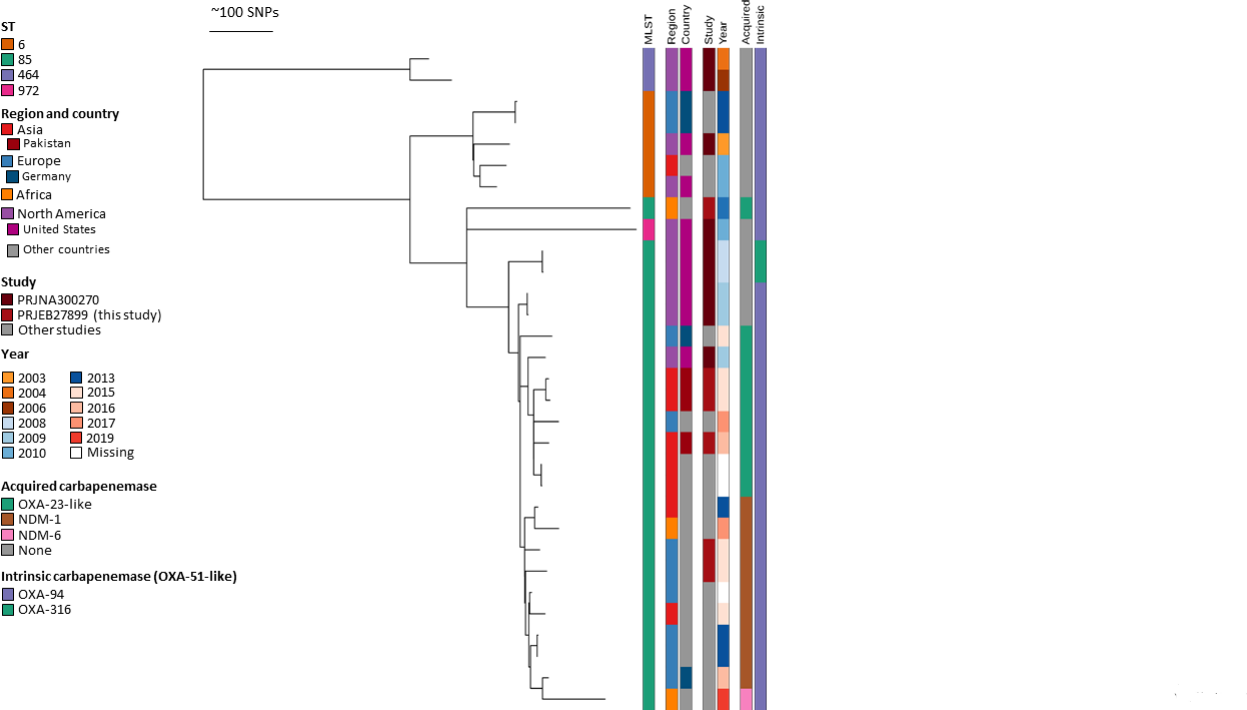
View of IC9. Whole genome maximum likelihood phylogenetic representation after recombination removal of the novel international clone 9 (IC9), based on six isolates from this study and 25 additional genomes from publicly available databases. The global scale of the isolates is visible; carbapenemases like OXA-23 and NDM-1 are widespread.

Since only six isolates of the novel IC9 were identified in this study (Fig. S4), twenty-five additional genomes from previous studies were included, confirming the international character of this clone (Fig. 5). The intrinsic *bla*_OXA-51-like_ variant in IC9 is *bla*_OXA-94_. Acquired oxacillinases (OXA-23) were found in 9/31 isolates, while MBLs NDM-1 and NDM-6 were found in 11 isolates, thus presenting a certain enrichment compared to other ICs, where there were only sporadic occurrences of MBLs.

## DISCUSSION

It is now over 10 years since the first study investigating a global collection of CRAB isolates (collected between 2004 and 2008) identified eight international clonal lineages IC1–IC8 (initially termed WW1–8) and provided a comprehensive insight into the dissemination of carbapenem resistance determinants within the CRAB population (17). Since then, the assignment to ICs has become an established criterion to classify *A. baumannii* isolates in an epidemiological context (14), and numerous studies have investigated the spread of ICs and resistance determinants in nosocomial CRAB isolates primarily at a local or national level (26–28).

With this study, we provide an update on the molecular epidemiology and distribution of carbapenemase genes within the worldwide CRAB population based on WGS of 313 CRAB isolates collected between 2012 and 2016 from 47 countries, spread over five geographical regions (Africa, Asia, Europe, Latin America, and North America). The Tigecycline Evaluation and Surveillance Trial (T.E.S.T.) isolates served as a valuable source for our study as they represent a worldwide collection of clinical bacterial isolates following a consistent protocol for sample inclusion and susceptibility testing (29). To obtain a comprehensive selection of CRAB isolates representing the participating countries in a well-balanced relation, the number of isolates included from each country corresponded to the population size of the participating countries. In this way, densely populated Asian countries, e.g., Thailand, were not underrepresented because of their limited number of study centers participating in the T.E.S.T. study and their lower number of collected isolates, and countries from Western Europe or the USA with many participating centers were not overrepresented. Given the well-known clonal population structure of *A. baumannii* and their tendency for hospital outbreaks and endemic persistence, every effort was made to enhance isolate heterogeneity and to exclude copy strains. To this end, the number of isolates included from a given study center was limited as much as possible and distributed over the entire study period. cgMLST analysis confirmed the heterogeneity of our study population with 296 isolates (94.6%) differing in at least one allele by cgMLST (data not shown). In this respect, cgMLST provided a simple measurement to assess the diversity of the collection. Whole genome SNP analysis, a more sophisticated approach, offered a higher resolution but is also more challenging with respect to data analysis. Both methods are comparable (Fig. S1 and 4), and their application may depend on the research question as well as the resolution required to answer that question.

About 94% of all isolates in our cohort were assigned to international clonal lineages, while less than 6% of isolates did not cluster with them, again emphasizing the clonal composition of the global CRAB population. In our study population, IC2 was by far the most frequent clonal lineage accounting for more than 62% overall, ranging from 10% in Latin America to 70% in North America and about 80% in Africa, Asia, and Europe. This is consistent with the findings from other studies on the epidemiology of CRAB (17, 30–33) and a substantial increase compared to our previous study, where IC2 represented only 49% of 492 investigated CRAB isolates (17).

In Latin America, in contrast, IC5 was the predominant clonal lineage accounting for 54% of CRAB isolates, while the clonal lineages IC1, IC2, and IC7 contributed approximately equally to the residual number of isolates (Fig. 1; Table 1). This confirms data from different Latin American countries indicating the predominance of *A. baumannii* clonal lineages IC4, IC5, and IC7 (often referred to as CC15^Pas^, CC79^Pas^, and CC25^Pas^, respectively) (32). For example, a high prevalence of IC4 was reported from Chile (33); in Brazil, IC5 was reported as the predominant clonal lineage, and the presence of IC7 is well documented in Bolivia (34, 35). It is obvious that the clonal distribution of CRAB in Latin America is different and deserves further research. From our expanded investigations into IC5 including sequences from published studies, we can see that this lineage has a distinct emergence of clades with particular geographic dominance. One clade is predominant in Brazil, characterized by ST79 with either *bla*_OXA-23_ or *bla*_OXA-72_, whereas the other Latin American clade is prevalent in Mexico, as described before (36), and has ST156 and the carbapenem resistance determinant *bla*_OXA-239_ (*bla*_OXA-23-like_). This clade seems to have emerged most recently (36) and also presents the most recent expansion within this extensive IC5 collection. The particular *bla*_OXA-239_ seems to be restricted to this geographic region and IC (36) and has not been found in other ICs in the larger Pathogenwatch database. The North American clade, on the other hand, is characterized by ST79 and *bla*_OXA-40_.

In our isolate collection, six isolates from Africa, Europe, and Asia harbored *bla*_OXA-94_ as their intrinsic *bla*_OXA-51-like_ variant and clustered with ST85^Pas^ (CC464^Pas^). These results indicate that there is a novel international clonal lineage “IC9” spread across three world regions, that in some cases was associated with the MBL *bla*_NDM-1_ (Fig. S4). In 2014, *A. baumannii* isolates carrying *bla*_OXA-94_ and NDM-1_,_ also belonging to ST85^Pas^, recovered from Syrian civil war victims, were first reported from Lebanon (37). Isolates harboring *bla*_OXA-94_ and NDM-1 were also reported from Spain, Saudi Arabia, and Tunisia as well as from other countries in our expanded analysis (Fig. 5), illustrating the challenge to contain the spread of novel multidrug-resistant CRAB clones in an era of globalization and mass migration (38–40).

Phylogenetic analysis of our collection of CRAB isolates showed that within single ICs, clusters of related isolates were mainly limited to one country, for instance, the clade of Brazilian IC5 (Fig. 4), probably representing local clones endemic to single institutions or with limited regional spread. However, in some cases, also, interregional clusters were found, which may be the result of patient transfer or tourism in our globalized world.

In our study cohort, resistance to carbapenems was mediated by acquired carbapenemase genes in 96% of isolates, and in the remaining 4% of isolates, carbapenem resistance was attributable to IS*Aba1* upstream of *bla*_OXA-51-like_ leading to overexpression of the intrinsic carbapenemase. Ten years ago, only 62.8% of CRAB worldwide possessed an acquired carbapenemase, while carbapenem resistance was mediated through IS*Aba1* upstream of *bla*_OXA-51-like_ in 37.2% of cases (17). Also, the distribution of carbapenemase variants among the acquired carbapenemases has changed over time. In our current collection of CRAB isolates, the percentages of *bla*_OXA-23-like_, *bla*_OXA-40-like_, and *bla*_OXA-58-like_ among all acquired carbapenemases were 78%, 18%, and 2%, respectively, whereas one decade ago, these percentages were 54%, 13%, and 36%, respectively (17). The shift from *bla*_OXA-58-like_ to *bla*_OXA-23-like_ has also been described in previous studies especially in Mediterranean countries and in China (41–43). The predominance of *bla*_OXA-23-like_ producing CRAB mainly representing IC2 has been reported worldwide (17, 26, 31).

Worldwide, reports of MBL-positive CRAB have increased during the last decade with *bla*_NDM_ reported from Africa, America, Asia, and Europe (11, 32, 44–46). Conversely, MBLs in our global collection were still rare at 2.2%, with *bla*_NDM_ observed in Africa and Europe only, while one isolate from the Philippines harbored *bla*_IMP_. For comparison, data from the global Pathogenwatch database indicate a slightly higher prevalence of MBLs (409/8,660 genomes, 4.7%); however, the sampling frame of the external studies is unknown, and sampling bias by inclusion of sequences from outbreak or endemic isolates is very likely. There appears to be an enrichment of MBLs in the novel IC9 compared to other ICs; however, with the small numbers currently included, this needs to be further explored. This obvious discrepancy of reporting on MBL-positive CRAB possibly reflects a publication bias overestimating the burden of MBLs in *A. baumannii*.

Our analysis of capsule KL and outer core OCL types supports previous studies that there is high diversity in the major immunogenic polysaccharide KL types with less diversity in OCL types (47). The poor correlation between KL, OCL, and IC is evident in this collection as well; however, we can contribute some advance to the global view and to the less well-described ICs and their KL and OCL diversity.

Our study has several limitations. All isolates investigated were obtained from the isolate collection of the international T.E.S.T. study, which, to our knowledge, represents the largest collection of contemporary *A. baumannii* isolates worldwide. Its original purpose was monitoring the *in vitro* activities of tigecycline and a panel of marketed and novel antimicrobials against clinically important Gram-positive and Gram-negative bacterial isolates (29). The T.E.S.T. study was not designed to reflect the true prevalence of CRAB or other bacterial species in the respective study centers, and the number of study centers among countries contributing to the T.E.S.T. study has varied substantially over the study period as has the number of isolates contributed by some centers. To control for this, the composition of the strain collection used for this study was primarily based on the population size of the participating countries. Also, the countries contributing study centers to the T.E.S.T. study represent only 25% of the global population, and many large and densely inhabited countries including China, India, Russia, and Turkey and the majority of African countries were not part of this study. To control for this, we added additional genomes from published studies retrieved from Pathogenwatch including genomes from China and India and used these data, in particular, for an expanded analysis of IC5, IC7, and IC9. A major limitation of this data set, however, is the largely unknown study designs, whether this be selection bias toward sampling of highly resistant isolates, isolates with particular resistance genes, outbreak investigations, environmental samples, or else.

In summary, our data suggest that the global CRAB population currently comprises at least nine clonal lineages that have disseminated worldwide, with IC9 identified as a novel clonal lineage. Isolates representing IC2 and harboring *bla*_OXA-23_ are by far predominant in most parts of the world, but the distribution of ICs and carbapenem resistance determinants can vary widely among different geographical regions with Latin America standing out. In particular, globalization, migration, and the use of antimicrobials will shape the CRAB population in the future and continue to challenge our healthcare systems worldwide.

## MATERIALS AND METHODS

### Bacterial isolates and antimicrobial susceptibility testing

The global T.E.S.T. is a worldwide surveillance study collecting Gram-positive and Gram-negative clinical bacterial isolates. Minimum inhibitory concentrations (MICs) of a panel of marketed antimicrobials were determined at the participating laboratories by broth microdilution following CLSI guidelines (29). Between 2012 and 2016, 3,295 meropenem-resistant *A. baumannii* clinical isolates were collected during this study. These isolates were obtained from hospitals in 47 countries representing a total population of around 2.2 billion people spread over five geographical regions, i.e., Africa, Asia, Europe, Latin America, and North America (Fig. 1B; Table S1). Among these, we selected approximately 1 isolate per 6.8 million inhabitants per country from the above-mentioned cohort to represent the participating countries equally and to exclude sampling bias resulting from some centers/countries contributing more isolates than others. This sampling strategy resulted in 326 non-copy *A. baumannii* isolates for further investigation. The calculated number of isolates per country was distributed equally among the participating study centers of each country. By limiting the number of isolates to approximately one isolate per center per year, isolates from local outbreaks representing the same strain type were largely excluded.

Species identification was confirmed by *gyrB* multiplex PCR and the presence of the intrinsic *bla*_OXA-51-like_ (48, 49). Carbapenem resistance was confirmed phenotypically by E-test (bioMérieux, Nürtingen, Germany) for meropenem and imipenem. Resulting MICs were interpreted according to current EUCAST breakpoints for *Acinetobacter* spp. (https://www.eucast.org/clinical_breakpoints/).

### Whole genome sequencing and analysis

Genomic DNA of all isolates was extracted using the MagAttract HMW DNA Kit (Qiagen, Hilden, Germany) following the manufacturer’s instructions and was aliquoted for WGS as well as to a series of multiplex PCRs described below. Sequencing libraries were prepared using the Nextera XT library prep kit (Illumina GmbH, Munich, Germany) for a 250-bp paired-end sequencing run on an Illumina MiSeq sequencer. The obtained reads were assembled *de novo* with use of the Velvet assembler integrated in the Ridom SeqSphere^+^ v.8.3.63 software (Ridom GmbH, Münster, Germany) and SPAdes 3.9 (25, 50). Reads were mapped to reference genomes of respective international clones using smalt (v0.7.6, www.sanger.ac.uk/science/tools/smalt-0) (IC1, A1 CP010781; IC2, 1656-2 CP001921; internal references for IC9, 1242655). Reads were filtered for quality, and single nucleotide polymorphisms, called when present in 75% of reads. For quality assurance, we set a minimum threshold of 90% core genome targets. The median value was 98.1% targets. Recombination was identified and removed by applying gubbins (51), and the resulting alignment was used for tree estimation using RAxML v8.2.12 with GTRGAMMA model and a random seed (52). Since we were unable to include CRAB isolates from some important countries such as China and India as these were not part of the T.E.S.T. study, we sought to close our knowledge gap by investigating 8,660 additional genome sequences available through Pathogenwatch (https://pathogen.watch) which had country information available (Table S5). Assemblies were downloaded in March 2023 through the website and included with our own assemblies in particular for an expanded analysis of IC5, IC7, and IC9. Genomes were annotated using Prokka v.12-beta (53), and core genes were identified using Roary v3.13.0 (54) with the default 95% identity cutoff and 99% core genes. SNP sites were extracted from the core gene alignment, and RAxML was used for tree estimation. Visualization of trees was carried out using iTOL (55) and with phandango for recombination (56). KL and O loci were determined using Kleborate (57). The raw sequencing reads generated in this project were submitted to the European Nucleotide Archive under the study accession number PRJEB27899 (Table S1).

### Molecular epidemiology

Sequence types according to the “Oxford” and the “Pasteur” seven-loci MLST schemes were derived from genome assemblies of all isolates using the pubMLST website (https://pubmlst.org/abaumannii) (18, 19). New alleles and STs were identified and submitted to the pubMLST database. Clonal complexes were defined as the “founder” ST (potential ancestral type) and its single-, double-, and triple-locus variants (SLVs, DLVs, and TLVs) applying the BURST function available at pubmlst.org. The variant of the intrinsic *bla*_OXA-51-like_ and the CCs derived from both schemes served as criteria to assign each isolate to the eight established ICs (17, 20, 21).

All CRAB isolates were further investigated applying a validated cgMLST scheme, including 2,390 target alleles, using the Ridom SeqSphere^+^ v. 8.3.63 software (Ridom GmbH) (25), to illustrate closer relationships of isolates within one IC, especially with interest in intra- and interregional clusters. Isolates differing in ≤9 alleles were considered closely related and designated a cluster of isolates.

### Identification of carbapenemases

The presence of OXA-encoding genes (*bla*_OXA-51-like_, *bla*_OXA-23-like_, *bla*_OXA-40-like_, *bla*_OXA-58-like_, *bla*_OXA-143-like_, and *bla*_OXA-235-like_) was initially investigated using a previously described multiplex PCR (6). Two further multiplex PCRs were applied to detect rarer carbapenemases found in *A. baumannii*, *bla*_GES_, *bla*_GIM_, *bla*_IMI_, *bla*_IMP_ *bla*_KPC_, *bla*_NDM_, *bla*_VIM_, and IS*Aba1* upstream of *bla*_OXA-51-like_ (ISAba1-*bla*_OXA-51-like_) (58). Additionally, genome assemblies were investigated using the CGE web-tool ResFinder 2.1 (https://cge.cbs.dtu.dk/services/ResFinder/) and with abricate v0.9.8 (https://github.com/tseemann/abricate) against the ResFinder database (59) to identify the distinct variants of carbapenemase families identified by PCR and confirmed by BLAST analysis of the amino acid sequence at the beta-lactamase database (http://www.bldb.eu/).

## Data Availability

The raw sequencing reads generated in this project were submitted to the European Nucleotide Archive under the study accession number PRJEB27899, with individual accession numbers listed in Table S1. Genomic data from other studies for ICs 5, 7, and 9 can be found in Tables S5 to S8.

## SUPPLEMENTAL MATERIAL

Figure S1 mbio.02260-23-s0001.tif Comparative view of IC5.

Figure S2 mbio.02260-23-s0002.tif IC1 recombinogenic regions.

Figure S3 mbio.02260-23-s0003.tif Phylogeny of IC7 based on 12 isolates from this study and additional 127 genomes from published databases.

Figure S4 mbio.02260-23-s0004.tif Comparative view of 6 isolates representing novel international clone 9 (IC9).

Supplemental Material mbio.02260-23-s0005.docx Figures S1 to S4; Tables S2 to S4; captions to other supplemental tables.

Table S1 mbio.02260-23-s0006.xlsx Metadata for isolates sequenced, including accession numbers, sequencing statistics, and isolation and typing information.

Table S5 mbio.02260-23-s0007.xlsx Metadata download for isolates from Pathogen.Watch (date: 03/2023).

Table S6 mbio.02260-23-s0008.xlsx Metadata IC5 for all isolates, including those from Pathogen.Watch.

Table S7 mbio.02260-23-s0009.xlsx Metadata IC7 for all isolates, including those from Pathogen.Watch.

Table S8 mbio.02260-23-s0010.xlsx Metadata IC9 for all isolates, including those from Pathogen.Watch.

ASM does not own the copyrights to Supplemental Material that may be linked to, or accessed through, an article. The authors have granted ASM a non-exclusive, world-wide license to publish the Supplemental Material files. Please contact the corresponding author directly for reuse.
