# Supplementary figures and images for: A global view on carbapenem-resistant Acinetobacter baumannii
Source: mBio. 2023 Oct 26;14(6):e02260-23. doi: 10.1128/mbio.02260-23 (PMC10746149; doi:10.1128/mbio.02260-23)

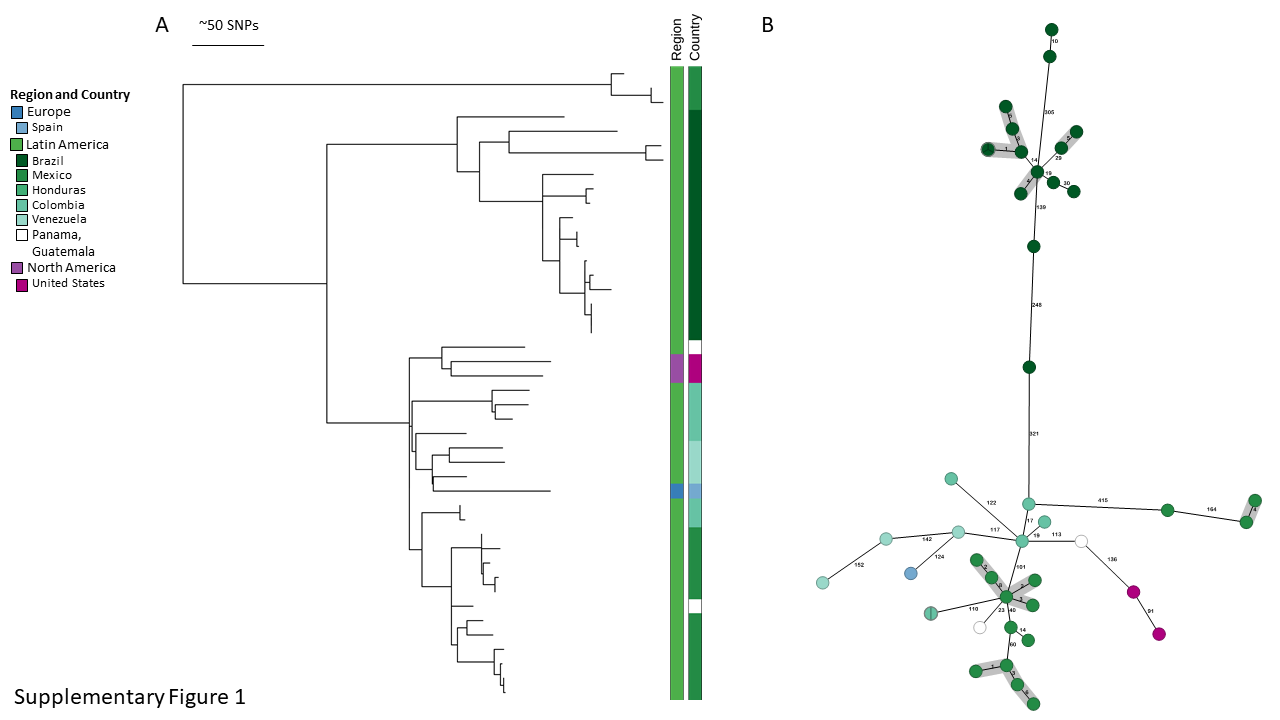

Supplement: Figure S1 — Comparative view of IC5. [file mbio.02260-23-s0001.tif]

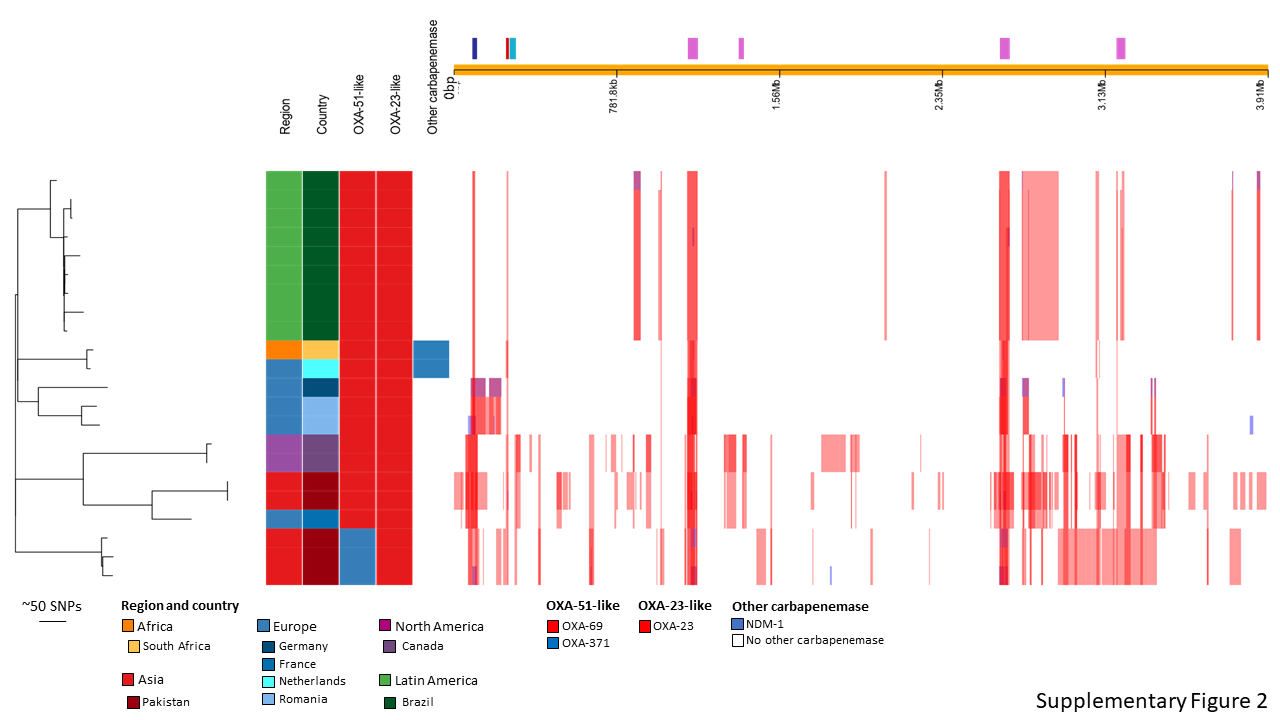

Supplement: Figure S2 — IC1 recombinogenic regions. [file mbio.02260-23-s0002.tif]

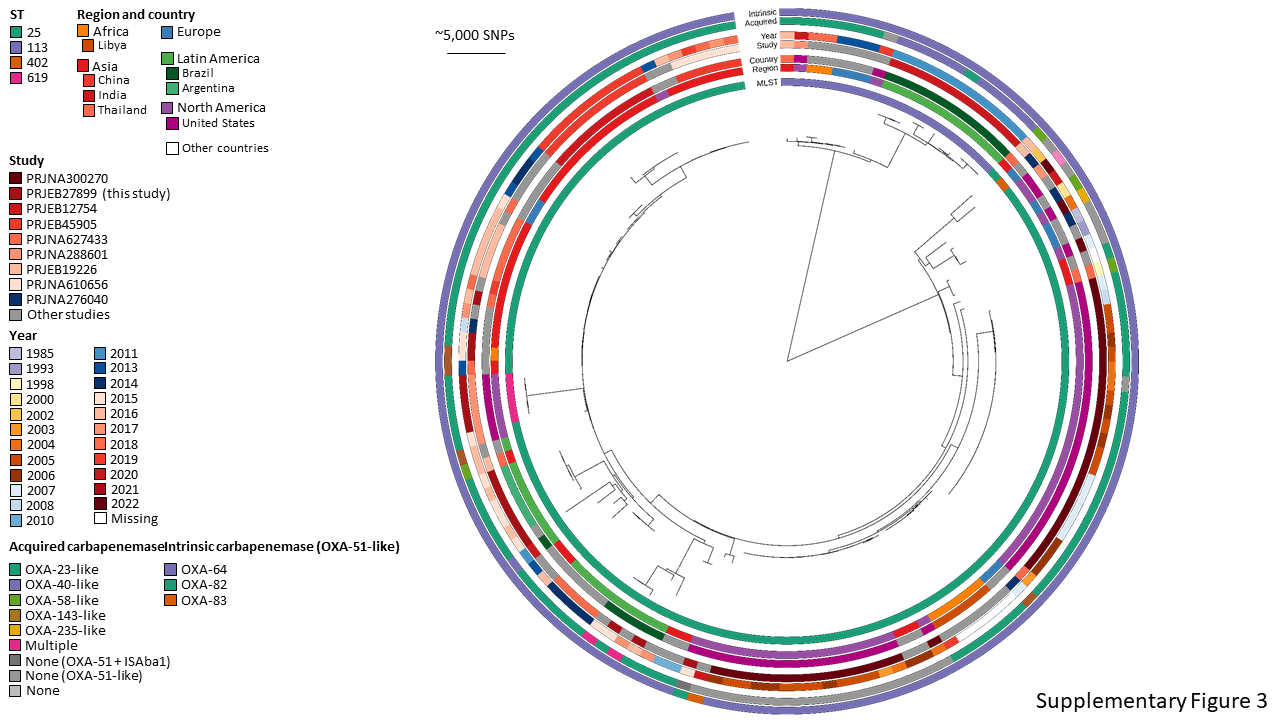

Supplement: Figure S3 — Phylogeny of IC7 based on 12 isolates from this study and additional 127 genomes from published databases. [file mbio.02260-23-s0003.tif]

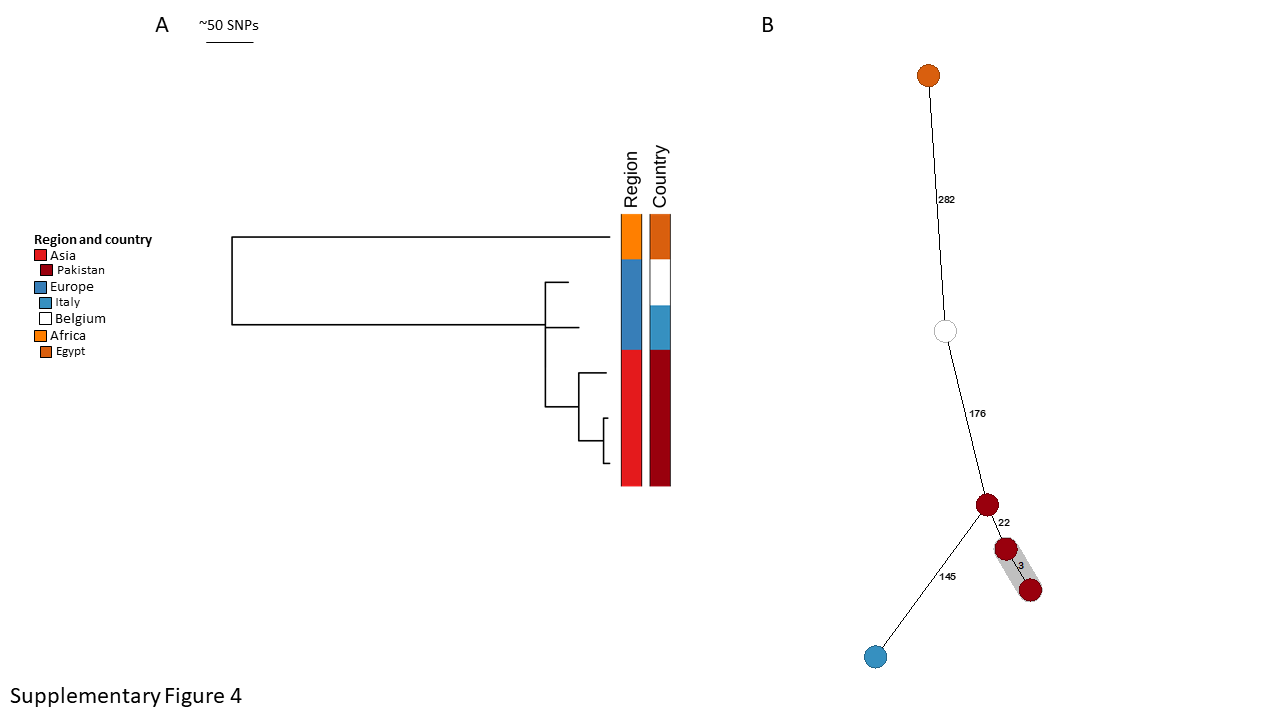

Supplement: Figure S4 — Comparative view of 6 isolates representing novel international clone 9 (IC9). [file mbio.02260-23-s0004.tif]
