# Supplementary material for: A global view on carbapenem-resistant Acinetobacter baumannii
Source: mBio. 2023 Oct 26;14(6):e02260-23. doi: 10.1128/mbio.02260-23 (PMC10746149; doi:10.1128/mbio.02260-23)
Supplement: Supplemental Material — Figures S1 to S4; Tables S2 to S4; captions to other supplemental tables. [file mbio.02260-23-s0005.docx]

**Supplementary Figures**

**Supplementary Figure S1:** Comparative view of IC5. **A.** Whole genome maximum likelihood phylogeny after recombination removal of Latin American IC5 based on 44 isolates from this study. **B.** Minimum spanning tree based on 2390 genes: *A. baumannii* cgMLST. Same country colour scheme used as in A. Same country clustering is apparent.

**Supplementary Figure S2:** IC1 recombinogenic regions. Note expansion of a sublineage in Brazil. Most isolates contained intrinsic *bla*_OXA-69_. Of the acquired carbapenemases apart from OXA-23, which was present in all isolates, NDM-1 was the only other carbapenemase detected. Recombination blocks (red) along the chromosome coordinates (top) are highlighted. Recombinogenic regions in part match with known regions of difference: KL1 capsule cluster (dark blue), MDR region (arsenic resistance, red; MARR, light blue), and phage (pink).

**Supplementary Figure S3**: Phylogeny of IC7 based on 12 isolates from this study and additional 127 genomes from published databases. Most isolates had intrinsic OXA-64 as the *bla*_OXA-51-like_ variant, and carried acquired OXA-23. The exception is one US isolate which carried OXA-82 as the *bla*_oxa-51-like_ variant and no OXA-23. Another isolate without OXA-23 harboured NDM-1 instead.

**Supplementary Figure 4:** Comparative view of 6 isolates representing novel international clone 9 (IC9). **A.** Whole genome maximum likelihood phylogeny after recombination removal. **B.** Minimum spanning tree generated using Ridom SeqSphere+. Each circle represents one individual isolate based on sequence analysis of 2.390 cgMLST target genes. Closely related genotypes (≤9 alleles difference) are shaded grey and designated one cluster. Same country colour scheme used as in A. Same country clustering is apparent, as is the close relationship between two isolates from Pakistan. The global scale of the isolates is visible.

**Supplementary Tables**

**Supplementary Table S1.** Metadata of isolates sequenced in this project: geographic information, clinical information, date of isolation, sequencing statistics, allocation to International Clones (IC), allocation to Pasteur and Oxford MLST schemes, minimum inhibitory information (MIC) to antibiotics tested, genotypic data for carbapenemase genes, capsule (K and OC locus) detected.

**Supplementary Table S2.** Regions and countries of origin of 313 carbapenem-resistant *A. baumannii* isolates

| Regions (no. of isolates/centres) | Participating countries (no. of isolates per country) |
| --- | --- |
| Africa (29/6) | Egypt (14), Morocco (5), South Africa (8), Tunisia (2) |
| Asia (88/15) | Hongkong (1), Jordan (1), Kuwait (1), Malaysia (5), Pakistan (26), Philippines (15), Saudi Arabia (5), Singapore (1), South Korea (8), Taiwan (4), Thailand (10), Vietnam (11) |
| Europe (67/56) | Austria (1), Belgium (2), Croatia (1), Czech Republic (1), Denmark (1), France (10), Germany (12), Greece (2), Hungary (2), Ireland (1), Italy (9), Latvia (1), Lithuania (1), Netherlands (2), Poland (6), Portugal (2), Romania (3), Spain (7), Sweden (1), Switzerland (1), United Kingdom (1) |
| Latin America (76/15) | Argentina (7), Brazil (32), Chile (3), Colombia (7), Guatemala (3), Mexico (18), Panama (1), Venezuela (5) |
| North America (53/22) | Canada (4), United States (49) |

**Supplementary Table S3.** Molecular characteristics and countries of origin of 313 carbapenem-resistant *A. baumannii* isolates

| IC | *bla*_OXA-51-like_ variants | CC^Ox^ | ST^Ox^ | CC^Pas^ | ST^Pas^ | Countries of origin |  |  |
| --- | --- | --- | --- | --- | --- | --- | --- | --- |
| IC1 | *bla*_OXA-69,_ *bla*_OXA-371_ | 231, 469 | 231, 387, 498, 1567, 1932 | 1 | 1, 81, 94,315, 1106 | BR, CA, DE, FR, NL, PK, RO, ZA | |  |
| IC2 | *bla*_OXA-66,_ *bla*_OXA-66*,_ *bla*_OXA-82*,_ *bla*_OXA-83*,_ *bla*_OXA-109*,_ *bla*_OXA-113*,_ *bla*_OXA-834_ | 208 | 136, 191, 195, 208, 218, 281, 348, 368, 369, 425, 436, 448, 450, 451, 452, 473, 493, 502, 539, 686, 784 787, 801, 804, 848, 1114, 1128, 1286, 1289, 1632, 1684, 1697, 1701, 1704, 1705, 1714, 1715, 1890, 1933, 1940, 1950, 1951 | 2 | 2, 45, 185, 187, 195, 571, 600, 604, 636, 641, 1160, 1312,  1313 | AR, AT, BE, BR, CA, CH, CZ, DE, DK, EG, ES, FR, GB, GR, HK, HR, HU, IT, JO, KR, KW, LT, LV, MA, MX, MY, NL, PH, PK, PL, PT, RO, SA, SE, SG, TH, TN, TW, US, VN, ZA | |  |
| IC3 | *bla*_OXA-71,_ *bla*_OXA-113*_ | 929 | 106, 203 | 1017 | 3, 229 | ES, US | |  |
| IC4 | *bla*_OXA-51,_ *bla*_OXA-219*_ | 103 | 225, 236 | 15 | 15, 318 | AR, CA, CL | |  |
| IC5 | *bla*_OXA-65,_ *bla*_OXA-65*,_ *bla*_OXA-117_ | 636 | 124, 205, 233, 758, 783, 924, 934, 1054, 1694, 1935 | 79 | 79, 156, 422, 730,  1163 | BR, CO, ES, GT, MX, PA, US, VE | |  |
| IC6 | *bla*_OXA-90_ | 944 | 944 | 78 | 78 | CO, GT | |  |
| IC7 | *bla*_OXA-64,_ *bla*_OXA-82*_ | 229 | 229, 690, 1489, 1760 | 25 | 25 | AR, BR, SA, TH, US, VE, ZA | |  |
| IC8 | *bla*_OXA-68_ | 447 | 447, 642 | 10 | 23, 10 | FR, MA, PK, PH | |  |
| IC9 | *bla*_OXA-94_ | 1078 | 1580, 1752, 1934, 1936 | 464 | 85 | BE, EG, IT, PK | |  |
| IC, international clone; * IS*Aba1* upstream of *bla*_OXA-51-like_; CC, clonal complex; ST, sequence type. Countries of origin: AR, Argentina; AT, Austria; BE, Belgium; BR, Brazil; CA, Canada; CH, Switzerland; CL, Chile; CO, Colombia; CZ, Czech Republic; DE, Germany; DK, Denmark; EG, Egypt; ES, Spain; FR, France; GB, United Kingdom; GR, Greece; GT, Guatemala; HK, Hongkong; HR, Croatia; HU, Hungary; IE, Ireland; IT, Italy; JO, Jordan; KR, South Korea; KW, Kuwait; LT, Lithuania; LV, Latvia; MA, Morocco; MX, Mexico; MY, Malaysia; NL, Netherlands; PA, Panama; PH, Philippines; PK, Pakistan; PL, Poland; PT, Portugal; RO, Romania; SA, Saudi Arabia; SE, Sweden; SG, Singapore; TH, Thailand; TN, Tunisia; TW, Taiwan; US, United States; VE, Venezuela; VN, Vietnam; ZA, South Africa. | | | | | | | | |

**Supplementary Table S4.** Geographical distribution of carbapenemases (OXAs and MBLs) among 313 carbapenem-resistant *A. baumannii* isolates

| Region (no. of isolates) | *bla*_OXA-23-like_ | *bla*_OXA-40-like_ | *bla*_OXA-58-like_ | *bla*_OXA-235-like_ | *bla*_NDM_ | *bla*_IMP_ | IS*Aba1*-*bla*_OXA-51-like*_ | | |
| --- | --- | --- | --- | --- | --- | --- | --- | --- | --- |
| Africa (29) ^a, b^ | 28 (96.6%) |  | 1 (3.4%) |  | 2 (6.9%) |  |  | | |
| Asia (88) ^c^ | 83 (94.3%) | 5 (5.7%) |  |  |  | 1 (1.1%) |  | | |
| Europe (67) ^d^ | 50 (74.6%) | 10 (14.9%) | 3 (4.5%) |  | 4 (6.0%) |  | 2 (3.0%) | | |
| Latin America (76) | 47 (61.9%) | 26 (34.2%) | 2 (2.6%) |  |  |  | 1 (1.3%) | | |
| North America (53) | 26 (49.0%) | 15 (28.3%) |  | 2 (3.8%) |  |  | 10 (18.9%) | | |
| Total (313) | 234 (74.8%) | 56 (17.9%) | 6 (1.9%) | 2 (0.6%) | 6 (1.9%) | 1 (0.3%) | 13 (4.2%) | | |
| OXAs, oxacillinases; MBLs, metallo-β-lactamases; ^a^ one isolate harboured *bla*_OXA-23_ and *bla*_NDM-1_; ^b^ one isolate harboured *bla*_OXA-23_ and *bla*_OXA-58_; ^c^ one isolate harboured *bla*_OXA-23_ and *bla*_OXA-72_; ^d^ two isolates harboured *bla*_OXA-23_ and *bla*_NDM-1_. _*_ IS*Aba1* upstream of *bla*_OXA-51-like_ as the only carbapenem resistance mechanism | | | | | | | |  |  |

**Supplementary Table S5.** Metadata download for isolates from Pathogen.Watch [date: 03/2023] and basic characterization of the collection (pivot tables: contributing countries, temporal distribution, international clones, carbapenemases; China: studies, temporal distribution, geographic distribution, international clone, carbapenemase; India: studies, temporatl distribution, international clone, carbapenemase).

**Supplementary Table S6.** Metadata IC5 for all isolates including those from Pathogen.Watch: accession numbers (study, run), geographic region, country, city/region, year, MLST, IC, combined carbapenemase, intrinsic carbapenemase, OXA-23-like, OXA-58-line, OXA-40-like, OXA-134-like, other carbapenemase, sequencing statistics (length, number of contic, largest contig, N50).

**Supplementary Table S7.** Metadata IC7 for all isolates including those from Pathogen.Watch: accession numbers (study, run), geographic region, country, city/region, year, MLST, IC, combined carbapenemase, intrinsic carbapenemase, OXA-23-like, OXA-58-line, OXA-40-like, OXA-134-like, other carbapenemase, sequencing statistics (length, number of contic, largest contig, N50).

**Supplementary Table S8.** Metadata IC9 for all isolates including those from Pathogen.Watch: accession numbers (study, run), geographic region, country, city/region, year, MLST, IC, combined carbapenemase, intrinsic carbapenemase, OXA-23-like, OXA-58-line, OXA-40-like, OXA-134-like, other carbapenemase, sequencing statistics (length, number of contic, largest contig, N50).
